# Maternal recall of breastfeeding duration twenty years after delivery

**DOI:** 10.1186/1471-2288-12-179

**Published:** 2012-11-23

**Authors:** Siv Tone Natland, Lene Frost Andersen, Tom Ivar Lund Nilsen, Siri Forsmo, Geir W Jacobsen

**Affiliations:** 1 Department of Public Health and General Practice, Norwegian University of Science and Technology, PO Box 8904 MTFS, , 7491, Trondheim, Norway; 2 Institute of Basic Medical Sciences, University of Oslo, Postboks 1046 Blindern, 0317, OSLO, Norway; 3 Department of Human Movement Science, Norwegian University of Science and Technology, Dragvoll, -7491, Trondheim, Norway

**Keywords:** Breastfeeding, Epidemiology, Long-term recall, Mothers, Validity

## Abstract

**Background:**

Studies on the health benefits from breastfeeding often rely on maternal recall of breastfeeding. Although short-term maternal recall has been found to be quite accurate, less is known about long-term accuracy. The objective of this study was to assess the accuracy of long-term maternal recall of breastfeeding duration.

**Methods:**

In a prospective study of pregnancy and birth outcome, detailed information on breastfeeding during the child’s first year of life was collected from a cohort of Norwegian women who gave birth in 1986–88. Among 374 of the participants, data on breastfeeding initiation and duration were compared to recalled data obtained from mailed questionnaires some 20 years later. Intraclass correlation coefficient (ICC), Bland-Altman plot, and Kappa statistics were used to assess the agreement between the two sources of data. Logistic regression was used to assess predictors of misreporting breastfeeding duration by more than one month.

**Results:**

Recorded and recalled breastfeeding duration were strongly correlated (ICC=0.82, *p* < 0.001). Nearly two thirds of women recalled their breastfeeding to within one month. Recall data showed a modest median overestimation of about 2 weeks. There were no apparent systematic discrepancies between the two sources of information, but recall error was predicted by the age when infants were introduced to another kind of milk. Across categories of breastfeeding, the overall weighted Kappa statistic showed an almost perfect agreement (κ = 0.85, 95% confidence interval [CI] 0.82 – 0.88).

**Conclusion:**

Breastfeeding duration was recalled quite accurately 20 years after mothers gave birth in a population where breastfeeding is common and its duration long.

## Background

The short-term health benefits from breastfeeding on mother and child are widely acknowledged [1]. Additionally, several studies have linked a history of breastfeeding with long-term maternal and child health outcomes, including reduced risk of type 2 diabetes [2], metabolic syndrome [3], hypertension and myocardial infarction [4] in mothers, as well as reduced risk of obesity [5] and type 2 diabetes [6], and lower blood pressure [7] and cholesterol levels [8] in children. Such studies have often relied on maternal recall of breastfeeding history, but, though this has been found to be an accurate estimate shortly after delivery [9,10], less is known about the long-term accuracy. Two previous studies have evaluated a recall interim of more than two decades, one of which comprised only college-educated women [11] while the other addressed a fairly small study sample [12]. Nevertheless, more data on the accuracy of recall are needed in order to enhance the interpretation of epidemiological findings. The objective of the current study was to assess the accuracy of maternal recall 20 years after delivery, and also to examine potential predictors of inaccurate reporting.

## Methods

We compared two sets of data that were collected from the same group of Norwegian women with a recall time varying from 20.2 to 22.5 years. First, breastfeeding data were collected prospectively in 1986–1989 by health professionals during the child’s first year of life, hereafter referred to as recorded breastfeeding data (our reference method). Second, recall of breastfeeding data was collected from a brief questionnaire mailed to the women in 2008, i.e. about 20 years after the birth of their child (our test method).

All mothers gave informed written consent. The study was approved by the Regional Committee for Medical and Health Research Ethics and by the Norwegian Data Inspectorate.

### Study population

Participants were selected from a population-based prospective observational study conducted in 1986–89 in the cities of Trondheim and Bergen, Norway, hereafter referred to as the parent study. The background and design of that study have previously been described in detail [13]. Briefly, it was designed to study the tendency among mothers to repeat patterns of fetal growth and birth outcomes in consecutive pregnancies. Caucasian women with singleton pregnancies and one or two previous births were included. Exclusion criteria were multiple pregnancy gestational age > 20 weeks at enrollment, and non-Caucasian ethnicity or language incompatibility.

The mother was screened and enrolled in the study around gestational week 17 based on referrals from her primary health care provider [13]. At the first visit her age (years), highest level of education, family status, smoking history, body height and pre-pregnancy weight were recorded. At birth, the gender, gestational age (days), birth weight (grams), length (cm) and head circumference (cm) of the offspring were recorded.

**Figure 1:**
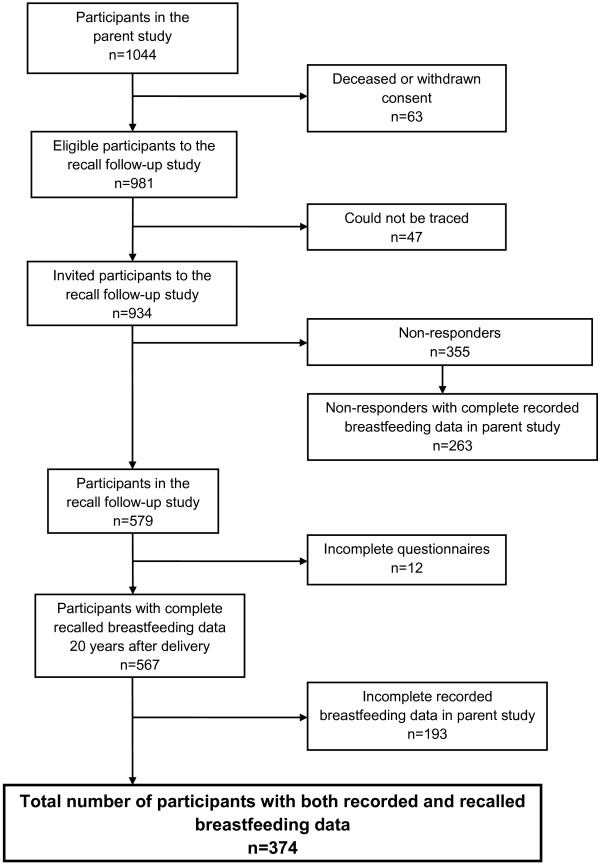
**Cohort profile.** Flow-chart of the participants in the parent and recall study of breastfeeding duration among Norwegian women.

Among 1,044 women who participated in the parent study, 63 women were deceased or had withdrawn their consent, which left a total of 981 women as eligible for our study (Figure 1). Among these, 47 women could not be traced. Hence 934 women were invited to participate in the recall study. A one page questionnaire was mailed to the mothers in 2008 some 20–22 years after delivery of her index child, i.e. the child enrolled in the parent study. The collected information included parity, the child’s birth weight, duration of breastfeeding in months, and the age when solid foods and other kinds of milk than breast milk were introduced.

A total of 579 women (participation rate 62.0%) returned the questionnaire, of whom 12 women were not included because they did not specify whether they had breastfed, or they confirmed that they had breastfed, but did not give any duration. For 193 of the 567 women who gave complete recall breastfeeding data, recorded breastfeeding data in the parent study were incomplete. In all of these cases, the records showed that the mother had breastfed at the first or several follow-ups, but there was no recorded cessation of breastfeeding. Thus, complete breastfeeding duration data from both the parent and recall studies were available for 374 women. Among the latter, 29 women were still breastfeeding at the final follow up 13 months after delivery. Our study is therefore based on 374 subjects with complete data from both sources in the analyses where we used breastfeeding duration as a categorical variable (Figure 1). When breastfeeding duration was employed as a continuous variable, the 29 women who were still breastfeeding at 13 months were not modeled since the exact duration extending 13 months was unknown. All the women in our study were known to have attempted to breastfeed.

Among the 355 women who were invited to the recall study, but who did not respond, 263 had complete breastfeeding data in the parent study. Data on breastfeeding duration and background maternal and child characteristics for the non-responders with complete recorded breastfeeding data are presented in Table 1.

**Table 1:** **Maternal and child characteristics of responders**, **non**-**responders and eligible participants**

**Variables**	**Responders**^**a**^**(n=374)**	**Non**-**responders**^**b**^**(n=263)**	***P***-**value**^**c**^	**Eligible participants (n=981)**
**Maternal characteristics**^**d**^				
Age at delivery, yrs, mean (SD)	29.1 (3.9)	28.7 (4.4)	0.327	29.1 (4.1)
Pre-pregnancy BMI, kg/m^2^, mean (SD)	21.2 (2.8)	21.2 (2.9)	0.949	21.1 (2.9)
College/ university education, n (%)	44 (26.5)	48 (18.3)	0.001	240 (24.5)
Never smokers, n (%)	108 (28.9)	42 (16.0)	<0.001	254 (25.9)
Married/cohabiting^e^, n (%)	330 (88.2)	224 (85.2)	0.076	856 (87.3)
No breastfeeding^f^, n (%)	0	1 (0.4)		1 (0.001)
Breastfeeding duration^f^ , months, median (IQR)	6.0 (6.0)	5.0 (5.0)	<0.001	6.0 (5.8)
**Child characteristics**, **index pregnancy**^**d**^				
Birth weight, g, mean (SD)	3440.7 (611.3)	3382.1 (595.4)	0.229	3424.1 (625.2)
Preterm birth of child, n (%)	15 (4.0)	14 (5.4)	0.434	62 (6.3)
SGA^g^, n (%)	64 (17.1)	52 (19.8)	0. 406	132 (13.6)
Gender,				
Boy, n (%)	167 (44.7)	140 (53.2)	0.033	490 (49.9)
Girl, n (%)	207 (55.3)	123 (46.8)		491 (50.1)
Birth order of index child,				
2^nd^ birth, n (%)	282 (75.4)	191 (72.6)	0.462	704 (71.8)
3^rd^ birth, n (%)	92 (24.6)	72 (27.4)		277 (28.2)
Age at introduction of cereals, months, median (IQR)^h^	4.0 (1.0)	4.0 (1.0)	0.140	4.0 (1.0)
Age at introduction of another kind of milk, months, median (IQR)^i^	5.0 (4.5)	2.5 (5.0)	<0.001	4.0 (4.8)

Abbreviations’: SD, standard deviation; BMI, body mass index; IQR, interquartile range; SGA, small for gestational age.

^a^ Responders with complete recorded breastfeeding data.

^b^ Non-responders with complete recorded breastfeeding data.

^c^*P*-value assessed using Chi-square test (categorical data) and independent samples t-test or the non-parametric Mann Whitney test (continuous data).

^d^ Background variables reported in parent study. Continuous variables presented as mean (SD) or median (IQR), categorical variables presented as n (%).

^e^ Number of women with unknown marital status: among responders: 39, among non-responders: 28

^f^ Number of women still breastfeeding at 13 months and therefore excluded in analysis of this variable: among responders: 29, among non-responders: 1. Number of missing data among eligible participants: 351.

^g^SGA defined as birth weight <10 percentile for gestational age, gender and parity [20]

^h^ Numbers with missing data on infant’s age at introduction of cereals: among responders: n=16, among non-responders: n=15. Number of missing data among eligible participants: 229.

^i^ Numbers with missing data on infant’s age at introduction of another kind of milk: among responders: n=36, among non-responders: n=19. Number of missing data among eligible participants: 261.

### Recorded breastfeeding data

In the parent study, mothers and children attended regular public health follow-ups in their respective communities at 6 weeks, 3 months, 6 months, 9 months and 13 months after delivery. During each visit the women were interviewed by a nurse whether they currently breastfed their baby (“Does the infant receive breast milk?” and “If the infant does not receive breast milk, at what age (of the infant) did you discontinue breastfeeding?”). If mothers had stopped breastfeeding, they reported the total duration in weeks at the first two visits and in whole months at the other three. The duration of any breastfeeding was defined as the total number of weeks or months the child received any breast milk, irrespective of the concomitant introduction of other fluids and solid foods. A breastfeeding data record was deemed complete if there was a recorded entry for non-breastfeeding or cessation of breastfeeding at any of the interviews. In case of several recorded entries for cessation of breastfeeding, the data from the follow-up closest to the child’s date of birth were used. The age of the infant when another kind of milk than breast milk and solid foods were introduced was also recorded.

### Recalled breastfeeding data

A total of 374 women with complete recorded breastfeeding data in the parent study returned the one page questionnaire. The questions about breastfeeding method and duration were as follows: “Did you breastfeed your son/daughter when he/she was a baby?” and “For how many months did you breastfeed?” We asked for the age of weaning in months because we anticipated that recall in weeks or days would be too inaccurate. Furthermore, breastfeeding duration is usually asked in months in recall epidemiological studies when breastfeeding duration is used as exposure [14,15]. In this paper, breastfeeding duration refers to any breastfeeding.

### Covariates

In some previous studies the accuracy of breastfeeding duration recall was associated with various maternal and child characteristics [9,11,12,16-19]. Based on those findings, we considered the following maternal covariates: age at study entry, pre-pregnancy body mass index (kg/m^2^; BMI), education (primary school, secondary school, college/university or unknown), and smoking status (ever vs. never smokers). Offspring covariates were birth weight, whether the newborn was preterm or small for gestational age (SGA; birth weight for gestation < 10^th^ percentile) [20], birth order and gender, and the age when the child was introduced to cereals and any other kind of milk than breast milk.

### Statistical analyses

The study outcome was the completed number of breastfeeding months. Given that one week is the approximate equivalent of Â¼ month; the initially reported weeks were recalculated into months by multiplying number of weeks by 0.25.

In order to evaluate the representativeness of the study sample, responders in the recall study were compared to non-responders using Chi-square statistics and independent samples *t*-test, or alternatively, the nonparametric Mann–Whitney test for variables that were not normally distributed, in which ties were split equally.

We calculated an intraclass correlation coefficient (ICC) between the recorded and recalled breastfeeding data, as a ratio of the variance between subjects over the total variance, in which an ICC over 0.75 was considered a strong correlation [21]. This absolute agreement coefficient was calculated overall and for subgroups defined by variables previously suggested as potentially associated with recall of breastfeeding in other populations. The Wilcoxon signed rank test was used to determine whether recalled breastfeeding duration varied significantly from that of the recorded data. To evaluate the possible relation between the discrepancies in breastfeeding duration in the recalled and recorded data, we used Bland-Altman plots [22]. This type of plot employs the difference between the two methods against their mean, and shows the magnitude of disagreement, spots outliers and investigates any possible relationship between the recall error and the recorded value.

Logistic regression was used to assess possible predictors of misreporting breastfeeding duration by more than a month. Variables that gave a *P*-value ≤0.10 in a simple model were further analysed in a multivariable model adjusting for potential confounders. A variable was considered a confounder if an odds ratio (OR) changed by 10% or more after adjustment, using the change-in-estimate method [23]. Variables that had no such effect were deleted from the model one by one in a stepwise manner. Since only 29 women in our study underreported their breastfeeding duration by more than a month, multivariable analyses to predict underreporting could not be performed.

Breastfeeding duration was grouped in a manner corresponding to a categorization scheme previously used in a study on maternal recall of breastfeeding duration [11]. However, since our sample consisted of women who were all known to have attempted breastfeeding, as well as a fairly large group of women who had breastfed for ≥13 months, we had to modify the categorization scheme. Our categories were 0, >0-3, 4 – 6, 7 – 9, 10 – 12 and ≥13 months. The aim was to assess the degree of misclassification by cross-tabulations. In order to evaluate agreement across categories, kappa statistics were calculated for each of the categories in relation to all of the other categories in 2×2 tables. For the ordinal multi-categories, we computed a quadratic weighted kappa, in order to attach greater emphasis to large differences between categories than small ones. Weighted kappa was given by the formula *K*_w_ = ∑ *wf*_*o*_*wf*_*c*_/*n*–∑ *wf*_*c*_, where *wf*_*o*_ = 1–(*ij*)^2^/(*k*–1)^2^, with *i* − *j* representing the difference between the row category on the scale and the column category on the scale (the number of categories of disagreement), for the cell concerned, and *k* representing the number of points on the scale [24,25]. Strength of agreement was evaluated according to Landis and Koch [26].

Analyses were performed with PASW (SPSS) for Windows version 18.0 (SPSS Inc., Chicago; IL, USA) and STATA software, release 11 for Windows (Stata Corp., College Station, TX, USA).

## Results

The 374 women were on average 29.1 years (SD 3.9) old at study entry (Table 1). Mean time since delivery was 21.2 years (SD 0.6) (data not shown). Four of five (80%) mothers delivered their second child (Table 1). Approximately one in four of the women had college or university education. According to data from the parent study, all women had attempted breastfeeding. Among non-responders, fewer had college/university education, fewer were never smokers, the mean breastfeeding duration was shorter, and another kind of milk (other than breast milk) was introduced earlier compared to the group of responders.

Almost two thirds (64%) of mothers recalled their breastfeeding duration to within one month compared to the recorded data (Table 2). Three times more women overreported breastfeeding than underreported it (n=95 vs. n=29, respectively, *p* < 0.001). Further, five out of six (83%) women recalled breastfeeding to within two months and 90% to within three (data not shown). Median breastfeeding duration among women who underreported by more than a month was 9.0 months (Interquartile range (IQR) 3.5), whereas women who overreported by more than a month had a median breastfeeding duration of 5 months (IQR 5.8) in the parent study. Among women who recalled their breastfeeding duration within a month, median breastfeeding was 6.0 months (IQR 6.0) (data not shown). Median difference between recalled and recorded breastfeeding duration among women who overreported it by more than a month was higher compared to women who underreported it (2.3 months [IQR 2.0] vs. 2.0 months [IQR 2.0] respectively, [*p*<0.001]) (data not shown).

**Table 2:** Recall error of breastfeeding duration by maternal and child characteristics (n=345)

		**Breastfeeding duration**		**Recall error**		
	**n**	**Recorded Median(IQR)**	**Recalled Median(IQR)**	**Underreporting > one month % (95%CI)**	**Overreporting > one month % (95%CI)**	**ICC**
**Maternal characteristics**						
Age at delivery (yrs)						
<25	46	3.0 (5.8)	4.0 (6.0)	10.9 (3.6, 23.6)	28.3 (16.0, 43.5)	0.76
25-<30	184	6.0 (5.0)	7.0 (5.0)	6.0 (3.0, 10.4)	29.4 (22.9, 36.5)	0.81
≥30	115	7.0 (5.0)	7.5 (5.0)	11.3 (6.2, 18.9)	24.4 (16.8, 33.2)	0.85
Pre-pregnancy BMI (kg/m^2^)^a^						
<25	314	6.0 (6.0)	7.0 (5.0)	8.3 (5.5, 11.9)	28.7 (23.7, 34.0)	0.81
≥25	30	3.5 (6.6)	4.5 (7.0)	10.0 (2.1, 26.5)	13.3 (3.8, 30.7)	0.93
Educational level						
Primary school	42	3.5 (6.3)	5.0 (5.0)	7.1 (1.5, 19.5)	33.3 (19.6, 49.6)	0.87
Secondary school	187	6.0 (5.0)	6.0 (5.0)	8.6 (5.0, 13.5)	28.3 (22.0, 35.4)	0.78
College/university	84	8.0 (4.0)	9.0 (4.0)	8.3 (3.4, 16.4)	22.6 (14.2, 33.1)	0.87
Missing information	32	8.0 (3.0)	8.5 (4.75)	9.4 (2.0, 25.0)	28.1 (13.8, 46.8)	0.74
Smoking status						
Never	90	8.0 (4.0)	9.0 (4.0)	8.9 (3.9, 16.8)	24.4 (16.0, 34.6)	0.82
Ever	255	6.0 (5.5)	6.0 (6.0)	8.2 (5.2, 12.3)	28.6 (23.2, 34.6)	0.81
**Child characteristics, index pregnancy**						
Birth weight (g)						
<3500	177	7.0 (6.0)	7.5 (5.0)	9.6 (5.7, 14.9)	28.8 (22.3, 36.1)	0.82
≥3500	168	6.0 (5.0)	7.0 (5.0)	7.1 (3.8, 12.1)	26.2 (19.7, 33.5)	0.83
Preterm birth of child						
Yes	11	8.0 (3.0)	10.0 (5.0)	n.c.^b^	4.5 (16.8, 76.6)	0.71
No	334	6.0 (6.0)	7.0 (5.0)	8.7 (5.9, 12.2)	27.0 (22.3, 32.1)	0.83
SGA^c^						
Yes	56	7.0 (5.0)	8.0 (5.5)	5.4 (11.2, 14.9)	39.3 (26.5, 53.3)	0.82
No	289	6.0 (6.0)	7.0 (5.0)	9.0 (6.0, 12.9)	25.3 (20.4, 30.7)	0.83
Gender,						
Male	156	6.0 (5.9)	7.5 (5.0)	9.0 (5.0, 14.6)	28.9 (21.9, 36.6)	0.80
Female	189	6.0 (5.0)	6.0 (5.0)	7.9 (4.5, 12.8)	26.5 (20.3, 33.4)	0.84
Birth order of index child,						
2^nd^ birth	269	6.0 (5.0)	7.0 (5.0)	7.8 (4.9, 11.7)	28.3 (23.0, 34.0)	0.82
3^rd^ birth	76	8.0 (6.5)	8.0 (3.75)	10.5 (4.7, 19.7)	25.0 (15.8, 36.3)	0.84
Age at introduction of cereals, months^d^						
< 4	96	4.0 (5.2)	5.0 (4.9)	10.4 (5.1, 18.3)	32.3 (23.1, 42.6)	0.78
≥4	236	7.0 (5.0)	8.0 (3.0)	7.2 (4.3, 11.3)	26.3 (20.8, 32.4)	0.82
Age at introduction of another kind of milk, months^e^						
< 4	132	2.5 (2.9)	3.0 (4.0)	3.8 (1.2, 8.6)	37.1 (28.9, 46.0)	0.69
≥4	191	8.0 (3.0)	8.0 (3.0)	10.5 (6.5, 15.7)	20.9 (15.4, 27.4)	0.67

Comparison of recalled and recorded breastfeeding data.

Abbreviations: IQR, interquartile range; ICC=Intraclass correlation coefficient; BMI, body mass index; SGA, small for gestational age.

^a^ Number with missing data body mass index: n=1.

^b^ n.c. =not calculated.

^c^ SGA defined as birth weight <10 percentile for gestational age, gender and parity [20].

^d^ Numbers with missing data on age at introduction of cereals: n=13.

^e^ Numbers with missing data on age at introduction of another kind of milk: n=22.

There was 97.9% agreement between maternal recall of their initial feeding practice (ever vs. no breastfeeding) and that recorded in the parent study 20 years earlier. Among the 39 women who had breastfed for less than 1.5 months according to the parent study, eight recalled that they had not breastfed (21%) their index child. Median breastfeeding duration was 6.0 (IQR 6.0) months in the parent and 7.0 (IQR 5.0) months in the recall study, respectively (data not shown). Median difference between recalled and recorded breastfeeding duration was 0.5 months (IQR 2.0, *p*<0.001). The overall intraclass correlation coefficient was high (ICC=0.82, *p*<0.001). Across subgroups of selected maternal and child characteristics, the agreement between recorded and recalled data on any breastfeeding duration varied from good to excellent, with the lowest value among those who introduced another kind of milk earlier than 4 months of infant’s age (ICC 0.69, *p*<0.001) and the highest ICC among women with a pre-pregnancy BMI ≥25 kg/m^2^ (ICC 0.93, *p*<0.001) (Table 2).

We also assessed possible predictors of overreporting breastfeeding duration by more than one month. Women who had breastfed 6 months or shorter in the parent study were more likely to overreport compared to those who had breastfed more than 6 months in the unadjusted analyses (OR 2.1; 95% CI 1.3, 3.4, *p*-value 0.003), but the results were no longer significant when introduction of another kind of milk and maternal pre-pregnancy BMI were added to the model (adjusted OR 1.2; 95% CI 0.6, 2.4). However, introduction of another kind of milk before the child was 4 months old remained significantly associated with overreporting by more than one month in the full model (adjusted OR 2.2, 95% CI 1.1, 4.2, *p*-value 0.022). No other variables were found to be independent predictors of overreporting.

**Figure 2:**
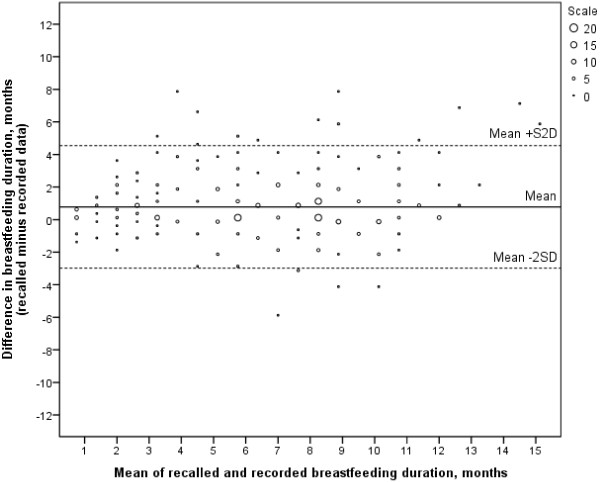
**Bland**-**Altman plot.** Differences between recalled and recorded breastfeeding duration vs. the mean of the two breastfeeding durations (n=345). Limits of agreement: Mean ±2 standard deviation (SD), 0.774 ± 2*1.882.

The Bland-Altman plot showed that most of the mean differences were positive, i.e. recalled breastfeeding duration tended to be overestimated, (Figure 2). The limits of agreement were wide and ranged from positive to negative values, implying that the women both under- and overestimated their breastfeeding duration in the recall follow-up study compared to the recorded data in the parent study. However, the plot did not indicate that the differences increased with an increase in breastfeeding duration. The plot also illustrated that the over- and underestimation were extreme in some of the cases. More specifically, 23 (6.7%) women had a difference in breastfeeding duration (recalled minus recorded duration) of more than the mean ±2SD.

### Breastfeeding duration by categories

Using the categories 0, >0-3, 4–6, 7–9, 10–12 and ≥13 months, breastfeeding duration was correctly classified by 245 (65.5%) women (Table 3). Another 113 (30.2%) women misclassified the duration by one category, whereas 16 women (5%) misclassified it by two or more (data not shown). The proportion of women who misclassified their breastfeeding duration was highest in the 4–6 months category (39.5%) and lowest in the ≥13 months category (17.2%) (Table 3). Our results indicate that fewer women in the three mid categories overreported, and more women underreported their breastfeeding duration as the recorded breastfeeding duration increased. Agreement was statistically significant (*p*<0.001) for all categories of breastfeeding duration, with a Kappa coefficient ranging between 0.47 and 0.72 (Table 3) for the separate categories. The overall weighted Kappa statistics across all of the categories was 0.85 (95% CI 0.0.82 – 0.89), which gives a ‘almost perfect’ strength of agreement [24].

**Table 3:** Breastfeeding duration by categories and distribution of recall error (n=374)

	**Recalled breastfeeding duration, months**								**Distribution of recall error, n (%)**		
**Recorded breastfeeding duration, months**	**Never**	>**0 - 3**	**4 - 6**	**7 - 9**	**10 -12**	≥**13**	**Total**	**ƙ**^**a**^	**Underreport**	**Exact recall**	**Overreport**
Never	0	0	0	0	0	0	0				
>0 - 3	8	63	22	6	0	0	99	0.70	8 (8.1)	63 (63.6)	28 (28.2)
4 - 6	0	3	49	24	4	1	81	0.47	3 (3.7)	49 (60.5)	29 (38.8)
7 - 9	0	0	13	72	20	3	108	0.50	13 (12.0)	72 (66.7)	23 (21.3)
10 -12	0	0	1	12	37	7	57	0.53	13 (22.8)	37 (64.9)	7 (12.3)
≥13	0	0	0	1	4	24	29	0.72	5 (17.2)	24 (82.6)	0 (0)
Total	8	66	85	115	65	35	374	0.85	42 (11.2)	245 (65.5)	87 (23.2)

^a^ Kappa statistics were calculated for each of the categories in relation to all of the other categories in 2×2 tables, as well as overall weighted ƙ for the ordinal multi categories of breastfeeding duration. All *p*-values <0.001.

## Discussion

Even with a median overestimation of about two weeks, this study among 374 Norwegian mothers showed that they recalled fairly accurately how long they breastfed their child after 20 years. A recall error of more than one month was explained only by the age of the child when another kind of milk was introduced. We found no statistically significant association between maternal education [9,12,16], gender of the child [12] or parity [12]. This is in agreement with previous studies and suggests that any lack of accuracy of maternal recall was non-differential. However, some comparisons may suffer from low statistical power that calls for caution in the interpretation of the results.

Recall was a fairly accurate measure of the mothers’ initial feeding method (ever versus no breastfeeding), with an agreement of 97.9%. Even among the 39 women who breastfed for less than six weeks (1.5 months) 4 out of 5 women had consistent results in both the recall and parent study. The eight women with inconsistent results recalled that they had not breastfed at all, while the records at 6 weeks follow-up examination indicated that they did so for a few weeks (range 1–5 weeks) after delivery. One may speculate that they had forgotten, mixed it up with another birth, or did not consider their brief period of breastfeeding duration important enough to mention in the recall study.

The accuracy of long-term (> 10 years) maternal recall has been investigated in a cohort of Jerusalem residents [12] and among Australian [16] and college-educated US women [11]. The sample size in all of these studies (n< 150) was small compared to the present one. Other authors have evaluated shorter term (≤10 years) recall [9,10,17,19,27-30]. Except for the study by Cupul-Uicab et al. [10], all reported fairly small sample sizes. Accordingly, we hold that we have conducted the first long term maternal recall study of breastfeeding duration by the use of a large population-based sample of women where breastfeeding was common and normally of long duration.

As in several previous studies, our median recalled breastfeeding duration was longer than in the health records from the parent study [12,16,18,19]. Nevertheless, our median difference between recalled and recorded breastfeeding duration was smaller than in comparable studies, even if the average recall period was the same or even longer [12,16]. Almost two thirds (64%) of our study women recalled their breastfeeding duration to within one month and 83% to within two months of the recorded duration in the parent study. Our accuracy was slightly less favorable than Eaton-Evans and Dugdale with a recall interval of three years (79% and 95% correct recall within one and two months, respectively) [9], but better than the one reported by Tienboon (35% and 59%, respectively) [16] after an interval of 15 years. Discrepancies of one month in any direction could be attributed to rounding errors, while larger discrepancies could possibly be explained by the mothers’ recall of breastfeeding duration of a different child. Yet we found no association with parity and misreporting breastfeeding duration by more than one month.

For comparison, the correlation coefficients in our study were slightly lower than reported in a study among Mexican women two to four years after delivery [10] and among Canadian women with a follow-up time of eight years [18], but higher than the report from college-educated US women with a recall period of more than 34 years [11].

There was some misclassification when breastfeeding duration was analysed in categories. Still, 95% of the women were either correctly classified or misclassified by only one category. The overall Kappa statistics of 0.85 suggested an almost perfect agreement [24], which is far higher than the findings of Promislow et al. showed [11]. Furthermore, the proportion that correctly classified breastfeeding duration was higher in our study (66% vs. 54%). Whereas the latter study comprised only college-educated women, ours consisted of women from all educational levels. Nevertheless, we found no association between education and misreporting by more than one month.

The fact that the highest proportion of misclassification was found in the mid categories may reflect floor and ceiling effects [31]. By design, women who breastfed for more than 13 months could not mathematically overreport duration. Correspondingly, women who breastfed for less than three months were unable to underreport, because of the categorization scheme we chose.

The strengths of our study are the comparably large sample of women from a population where breastfeeding is the accepted norm and of long duration, and the reasonably high response rate (62%). Another strength is the long-term recall period and the prospective standardised recording of breastfeeding data by health professionals. And whereas some previous studies have presented their findings as categorical data only [17,32], we report our outcome as both continuous and categorical variables.

One limitation of our study is the slightly different background characteristics of the responders as compared with the non-responders. Hence we cannot rule out the possibility that responders were more likely to recall their breastfeeding practices. Added to this, even though the response rate was reasonably high, we could not include all of the responders in our analyses because of missing data on recorded breastfeeding in the parent study. Hence our sample may not be fully representative for the general Norwegian population.

By design, the parent study did not include primiparae [13], which may be considered a second limitation. Breastfeeding duration from one infant to the next tends to be correlated [33], and therefore, multiparous women, who have breastfed two or more children for similar lengths of time, may be more likely to report it accurately than women with only one child. Third, participation in the parent study implied that the women attended some additional health check-ups during pregnancy and the child’s first year [13]. Therefore, we cannot rule out that our participants were more health conscientious, had a stronger intention to breastfeed and were more focused on their pregnancy and post-partum period. Fourth, almost one third of the responders in the recall study had incomplete data on breastfeeding duration in the parent study. More specifically, while the records of the parent study showed that the mothers had breastfed at one or more follow-up visits, the exact cessation of breastfeeding was not recorded. Among these, there were fewer children (4.7%) who were born small for gestational age [20]. One may speculate whether the public health nurse recorded the cessation date more scrupulously among children who were small for gestational age. Yet, it is unclear if and how this would affect the long-term maternal recall many years later. Fifth, among the responders in the recall study with both recorded and recalled breastfeeding data, there was a higher proportion of mothers of children classified as small for gestational age (26%). However, additional analyses did not indicate that mothers of children that were small for gestational age recalled breastfeeding duration more accurately than mothers of children that were not. Finally, breastfeeding has generally been the norm in Norway where more than 90% have breastfed for at least one week since the late 1960s [34]. Thus, breastfeeding initiation was probably close to 100% during the time of the parent study. It may be held that our results are not entirely applicable in populations where breastfeeding rates are lower. Even so, and in view of any purported selection of our study population, the high agreement between recalled and recorded breastfeeding duration supports the use of recalled breastfeeding duration as an exposure variable in epidemiological studies on maternal adverse health outcomes in later life, as has been done in a recent Norwegian study [35].

## Conclusions

The results of this Norwegian recall study among mothers 20 years after delivery show that their recall was fairly accurate in terms of the initial feeding method of their child and breastfeeding duration. Generalising our results to other populations with different breastfeeding behaviour may, however, not be entirely appropriate. Further studies should examine the potential effect of misreporting of breastfeeding duration on estimates of associated and later health outcomes.

## Competing interests

The authors declare that they have no competing interests.

## Authors’ contribution

STN conceived the idea, planned and collected the data in the present study, did the analyses and wrote the paper. GWJ headed the management of data collection in the parent study and participated in the planning of the present study. SF, LFA, TILN and GWJ participated in the analyses, interpreted the results and wrote the paper. All authors read and approved the final manuscript.

## Pre-publication history

The pre-publication history for this paper can be accessed here:

http://www.biomedcentral.com/1471-2288/12/179/prepub
